# Dual roles of the transmembrane protein p23/TMP21 in the modulation of amyloid precursor protein metabolism

**DOI:** 10.1186/1750-1326-2-4

**Published:** 2007-02-08

**Authors:** Kulandaivelu S Vetrivel, Ping Gong, James W Bowen, Haipeng Cheng, Ying Chen, Meghan Carter, Phuong D Nguyen, Lisa Placanica, Felix T Wieland, Yue-Ming Li, Maria Z Kounnas, Gopal Thinakaran

**Affiliations:** 1 Departments of Neurobiology and Neurology, The University of Chicago, Chicago, IL 60637, USA; 2 Committee on Neurobiology, The University of Chicago, Chicago, IL 60637, USA; 3 TorreyPines Therapeutics, Inc. La Jolla, CA 92037, USA; 4 Molecular Pharmacology and Chemistry Program, Memorial Sloan-Kettering Cancer Center, New York, NY 10021, USA; 5 Biochemie-Zentrum der Universitat Heidelberg, Im Neuenheimer Feld 328, D-69120 Heidelberg, Germany

## Abstract

**Background:**

Alzheimer's disease (AD) is characterized by cerebral deposition of β-amyloid (Aβ) peptides. Aβ is released from ectodomain cleaved amyloid precursor protein (APP) via intramembranous proteolysis by γ-secretase, a complex consisting of presenilin and a few other proteins. p23/TMP21, a member of the p24 family type I transmembrane proteins, was recently identified as a presenilin complex component capable of modulating γ-secretase cleavage. The p24 family proteins form oligomeric complexes and regulate vesicular trafficking in the early secretory pathway, but their role in APP trafficking has not been investigated.

**Results:**

Here, we report that siRNA-mediated depletion of p23 in N2a neuroblastoma and HeLa cells produces concomitant knockdown of additional p24 family proteins and increases secretion of sAPP. Furthermore, intact cell and cell-free Aβ production increases following p23 knockdown, similar to data reported earlier using HEK293 cells. However, we find that p23 is not present in mature γ-secretase complexes isolated using an active-site γ-secretase inhibitor. Depletion of p23 and expression of a familial AD-linked PS1 mutant have additive effects on Aβ_42 _production. Knockdown of p23 expression confers biosynthetic stability to nascent APP, allowing its efficient maturation and surface accumulation. Moreover, immunoisolation analyses show decrease in co-residence of APP and the APP adaptor Mint3. Thus, multiple lines of evidence indicate that p23 function influences APP trafficking and sAPP release independent of its reported role in γ-secretase modulation.

**Conclusion:**

These data assign significance to p24 family proteins in regulating APP trafficking in the continuum of bidirectional transport between the ER and Golgi, and ascribe new relevance to the regulation of early trafficking in AD pathogenesis.

## Background

Amyloid precursor protein (APP) is a type I membrane protein that is trafficked through the secretory and endocytic pathways in neuronal and non-neuronal cells, and is the precursor to 40–42 amino acid residue β-amyloid peptides (Aβ). Cerebral deposition of Aβ in senile plaques is a pathological feature of patients with Alzheimer's disease (AD), and Aβ deposits are also found in aged individuals. Aβ is liberated from APP via sequential proteolysis by β- and γ-secretases [1]. Cleavage of APP within the lumenal domain by BACE1, the major neuronal β-secretase, releases the APP ectodomain and generates the N-terminus of Aβ [2]. The APP ectodomain can also be released by cleavage at the "α-secretase" site within the Aβ domain by zinc metallopreotases such as TACE/ADAM17, ADAM9, ADAM10 and MDC-9, and an aspartyl protease BACE2 [3]. The C-terminal APP stubs (APP CTFs) resulting from α – and β-secretase cleavage serve as substrates for intramembranous proteolysis by γ-secretase, a multimeric complex made of presenilin (PS) 1 or 2, nicastrin, APH1 and PEN2 [4]. Mature components of the γ-secretase complex are found, and shown to be enzymatically active, at the cell surface as well as in multiple organelles such as the ER/Golgi intermediate compartment (ERGIC), Golgi apparatus, trans-Golgi network (TGN), and late endosomes [5]. Activation of Notch signaling also involves sequential proteolytic processing, which closely resembles proteolysis of APP. Following ligand binding at the cell surface, Notch is endocytosed and sequentially cleaved by ADAM family metalloproteases and γ-secretase [6]. Enhancer/suppressor screen studies in *Caenorhabditis elegans *originally suggested a role for p24 proteins in the transport regulation of Notch receptors to the cell surface [7]. Reducing the activity of the p24 family member SEL-9 increased the cell surface accumulation of a transport-defective GLP-1 mutant, and increased the activity of mutant LIN-12 or GLP-1. Recently γ-secretase complex was found to contain p23 (also called TMP21), a p24 family protein. Intriguingly, reducing p23 expression resulted in increased γ-secretase cleavage of APP, without affecting the proteolysis of Notch [8].

The p24 proteins are a phylogenetically-conserved family of type I transmembrane proteins [9] that are highly enriched in the ER, Golgi, and coat protein (COP) I and II transport vesicles [10,11]. Mammalian p24 family consists of six members, p23/TMP21, p24/p24a, p25/gp25L, p26/p24b, p27, and tp24, which function as hetero-oligomeric complexes [12]. Sorting motifs in the cytosolic tail of p24 proteins bind to coat proteins of COPI and COPII vesicles [13-16], and ADP-ribosylation factor 1 (ARF1) [17]. Furthermore, p23, p24a, and p25 are present in complexes with the Golgi reassembly stacking proteins GRASP55 and GRASP65 [18]. A yeast strain lacking all eight members of the p24 family was viable displaying only minor secretory deficits [19], while in mice targeted disruption of both p23 alleles resulted in early embryonic lethality [20]. Proposed roles of the p24 proteins include COP vesicle cargo receptors, regulators of COP vesicle budding, ER quality control, and organization of the Golgi apparatus [7,13,16,21-25]. Recent evidence suggests that p24 proteins may specifically populate a subset of COPI vesicles [26] or influence the formation of tubular transport intermediates [27]. Thus, their precise role in early secretory pathway trafficking, and more importantly sorting of specific cargo, still remains elusive.

Here, we investigated p23 modulation of APP metabolism and report that diminution of p23 expression in non-neuronal and neuroblastoma cell lines leads to increased biosynthetic stability and maturation of nascent APP, cell surface accumulation of APP, as well as secretion of sAPP. Selective increase in Aβ_42 _production associated with a familial AD (FAD)-linked PS mutation occurs independently of p23 modulation of APP metabolism. Finally, immunoisolation and immunofluorescence analysis reveals redistribution of APP and its adaptor Mint3/X11γ in cells with knockdown of p23 expression, suggesting a potential mechanism by which p23 may influence APP trafficking and sAPP release. Our data reveal a novel role for the p24 family proteins in regulating early secretory pathway trafficking of holo APP in addition to the recently reported modulation of γ-secretase cleavage of APP CTFs.

## Results

### siRNA knockdown of p23 expression affects secretion of APPs and Aβ peptides in mouse neuroblastoma cells

**Figure 1:**
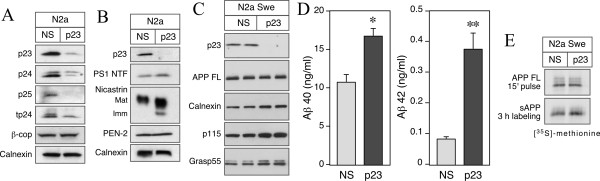
**siRNA-mediated depletion of p23 in N2a cells affects APP metabolism**. *A *and *B*, Lysates of N2a cells transfected with control non-specific (*NS*) or p23 siRNA were probed with antibodies specific for four p24 family members, three components of the γ-secretase, β-COP, and calnexin. Knockdown of p23 expression causes co-ordinate diminution the steady-state levels of p24, p25 and tp24. Also note the increased accumulation of immature and mature nicastrin in p23-depleted cells. *C*, Lysates of siRNA transfected N2a*Swe *cells were probed with antibodies specific for APP, calnexin, p115, and Grasp55. *D*, Secreted Aβ_40 _and Aβ_42 _in the media conditioned by N2a*Swe *were quantified using two-site ELISAs. The data represent mean ± SEM of three sets of transfections. * *p *< 0.05; ** *p *< 0.01. E, N2a*Swe *cells were labeled with [^35^S]met/cys for 15 min and APP FL were immunoprecipitated from cell lysates using Ab 369 (top); sAPP were immunoprecipitated using mAb P2-1 from media of cells labeled for 3 h (bottom).

Transfection with synthetic p23 siRNA markedly attenuated p23 expression (>85% knockdown of p23 expression) in mouse N2a neuroblastoma cells relative to non-specific control siRNA transfection (Fig. 1A). Previously based on deletion analysis in yeast and mammalian cells, it was reported that members of the p24 family depend on each other for proper assembly into stable oligomeric complexes [20,28]. Consistent with this prediction and a recent report [8], p23 knockdown produced concomitant reduction in the levels of p24, p25, and tp24. The steady-state levels of the ER resident transmembrane protein calnexin, Golgi associated β-COP subunit of the coatomer, and Golgi matrix proteins p115 and Grasp55 were unaffected by p23 knockdown (Fig. 1). However, we noted minor increase in PS1 NTF as well as an increase in the levels of immature and mature nicastrin, but the levels of PEN2 remained unchanged. In accordance with the recent report by Chen et al. [8], siRNA knockdown of p23 expression in N2a cells stably expressing an FAD-linked APP mutant (N2a*Swe *cells) increased secretion of Aβ_40 _by 55% (10.7 ng/ml in NS siRNA cells *versus *16.7 ng/ml in p23 siRNA cells; *p *< 0.05) and Aβ_42 _by approximately 4.5-fold (0.08 ng/ml in NS siRNA cells *versus *0.38 ng/ml in p23 siRNA cells; *p *< 0.01) as determined by ELISA of conditioned media (Fig. 1D). Thus both the total amount of fibrillogenic Aβ_42 _and the fraction of Aβ_42 _as a percent of total Aβ increased by siRNA depletion of p23 expression. Next we sought to determine whether secretion of sAPP also parallels increased secretion of Aβ in p23-depleted cells. For this analysis, we immunoprecipitated sAPP from the conditioned media of cells labeled for 3 h with [^35^S]met/cys and normalized sAPP levels to APP synthesis determined by 15 min pulse labeling. By phosphorimager quantification, we observed a 2-fold increase in the levels of sAPP secreted by N2a*Swe *cells transfected with p23 siRNA (Fig 1E). Our results are in accordance with the earlier reported role for p23 in modulating Aβ production, but since p23 knockdown invariably attenuates the expression of several p24 family proteins, our results also suggest a broader role for p24 family proteins in regulating APP metabolism and secretion.

### Knockdown of p23 affects APP maturation and cell surface expression

**Figure 2:**
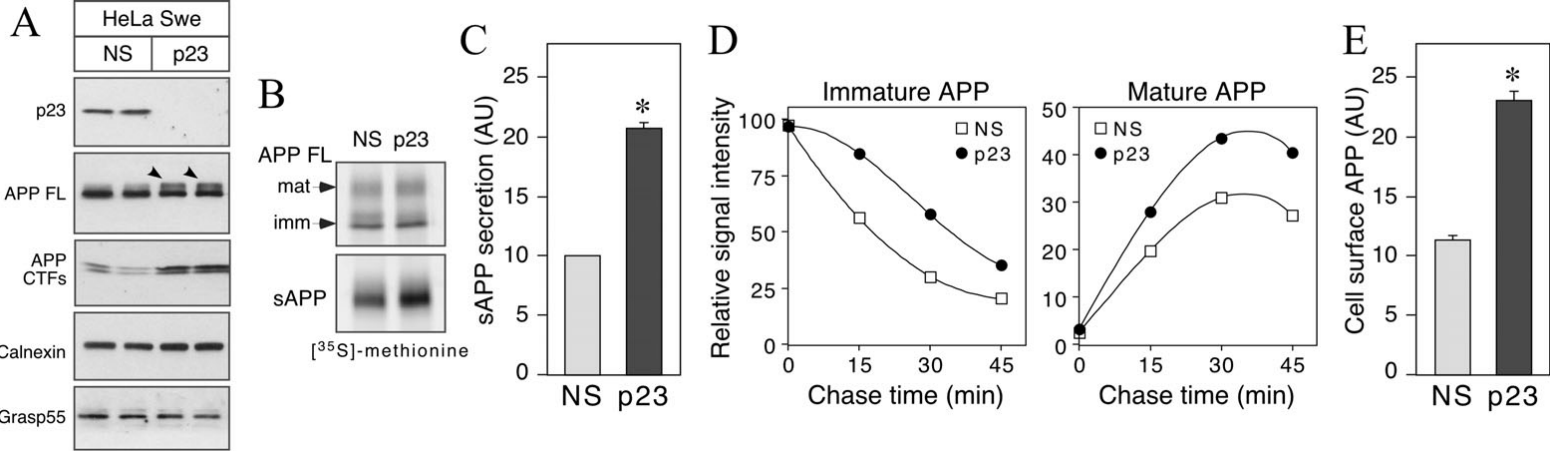
**p23 depletion affects APP maturation and secretion in HeLa cells**. *A*, Lysates of HeLa cells transfected with siRNA were probed with antibodies specific for p23, APP, calnexin, and Grasp55. *Arrowheads *indicate mature glycosylated APP. *B*, HeLa*Swe *cells were labeled with [^35^S]met/cys for 3 h and APP FL and sAPP were immunoprecipitated from cell lysates and conditioned media, respectively. *C*, The levels of sAPP in 3 h-labeled conditioned media were quantified and normalized to APP synthesis determined by 15 min pulse labeling. *AU*, arbitrary units. *D*, HeLa*Swe *cells were pulse-labeled for 15 min and chased for the times indicated. APP FL was immunoprecipitated from cell lysates and signal intensities of immature and mature APP at each time point were quantified by phosphorimaging and normalized to APP synthesis. *E*, The levels of cell surface APP were quantified by mAb P2-1 binding of live cells as descried under Methods. The graphs in *C *and *E *represent mean ± SEM of three sets of transfections (*p *< 0.001), and *D *represents the mean of two experiments.

Compared with the results from HEK293 cells [8], an increase in sAPP release observed in N2a neuroblastoma cells was unexpected. Furthermore, modulation of γ-secretase activity by p23 interaction with the presenilin complex seems insufficient to explain this perplexing observation on sAPP release, which is dependent on cleavage by α – or β-secretase. This prompted us to further examine p23 modulation of sAPP secretion in HeLa cells, as an additional non-neuronal cell line. Similar to N2a cells, transient siRNA knockdown of p23 expression in HeLa cells stably expressing an FAD-linked APP mutant (HeLa*Swe*) also markedly reduced the levels of other p24 family proteins without discernable effects on the levels of PS1, nicastrin, PEN2, β-COP or calnexin (Fig. 2A and data not shown). However, in p23-depleted cells we noted a clear increase in mature APP (upper band of the doublet), and APP CTFs resulting from cleavage of APP by α – or β-secretases. Continuous 3 h labeling using [^35^S]met/cys followed by immunoprecipitation analysis revealed a 2-fold increase (*p *< 0.001) in the levels of sAPP released into the media conditioned by p23-depleted cells, despite similar steady-state levels of immature and mature APP holoprotein relative to non-specific siRNA transfected cells (Fig. 2B and 2C).

**Figure 3:**
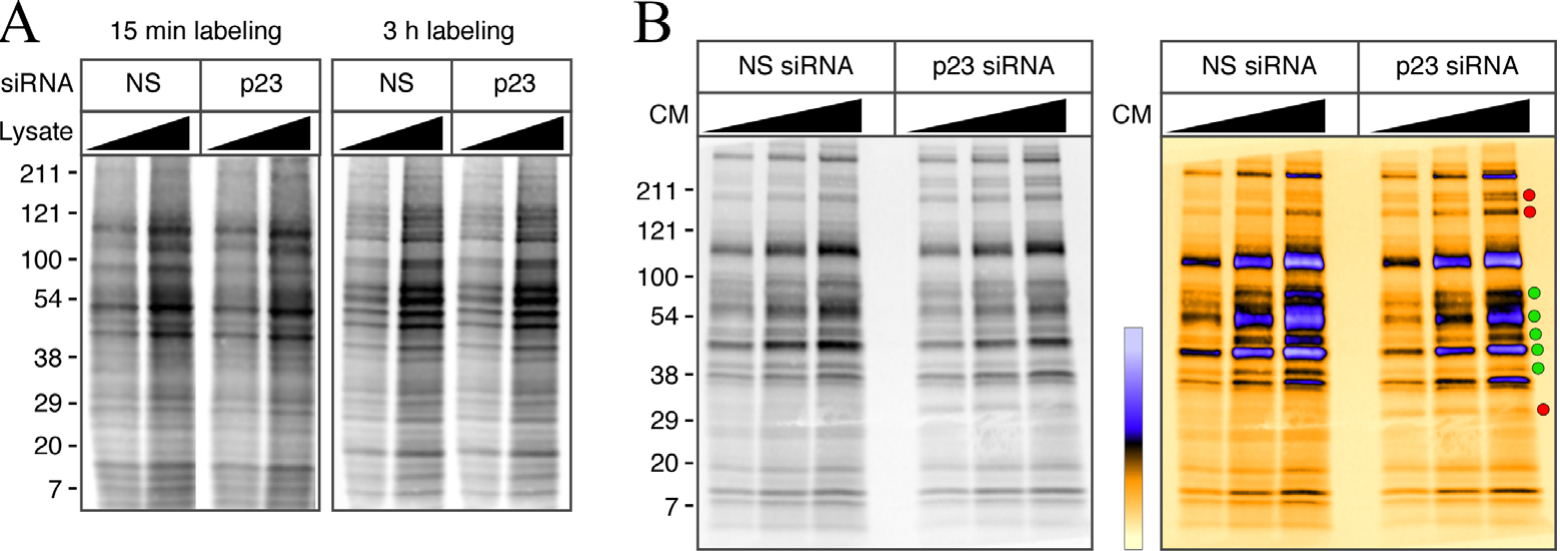
**p23 depletion has minor effects on overall protein secretion**. *A*, N2a*Swe *cells transfected with siRNA were labeled with [^35^S]met/cys for 15 min or 3 h. Aliquots of lysates (5 and 10 μl) were separated by SDS-PAGE and analyzed by phosphorimaging. *B*, 5, 10 and 20 μl of aliquots of conditioned media (CM) from cells labeled for 3 h were analyzed. In the panel on the right, the gray scale image is depicted in pseudocolor. Red and green circles indicate increase and decrease in the intensity of secreted polypeptides, respectively, in cells transfected with p23 siRNA relative to NS siRNA.

Does p23 knockdown alter the kinetics of nascent APP polypeptide maturation? To address this issue, we performed pulse/chase-labeling experiments. Although APP synthesis is similar to control cells following 15 min pulse labeling with [^35^S]met/cys, we found higher levels of immature and mature APP during the chase period in cells transfected with p23 siRNA (Fig. 2D). These data suggest that nascent core-glycosylated APP polypeptides are relatively more stable, and readily undergo complex glycosylation in cells with attenuated expression of p24 proteins. Increased maturation of APP as well as sAPP secretion, suggested the possibility that loss of p23 expression might enhance APP trafficking to the cell surface. Indeed, quantitative analysis revealed a 2-fold increase (*p *< 0.001) in the steady-state levels of cell surface APP in cells transfected with p23 siRNA (Fig. 2E). Next, we considered the possibility that p23 depletion and diminution of additional p24 family proteins might cause a general increase in cellular secretion. To test this idea, we assessed total protein synthesis and secretion in p23 depleted cells. SDS-PAGE analysis of detergent lysates prepared from control and p23 siRNA transfected cells showed similar levels of total protein synthesis following 15 min pulse labeling or 3 h continuous labeling with [^35^S]met/cys (Fig. 3A). However, SDS-PAGE analysis of conditioned medium showed decrease in the levels of several secreted polypeptides and small increase in a few polypeptides (Fig. 3B). Thus, depletion of p24 family members does not affect overall protein synthesis, but causes a small decrease in the secretory potential of the cell. On the other hand, the results described above suggest that p23 plays a role in negatively regulating APP maturation and trafficking, in addition to the reported role in γ-secretase processing.

### Stable knockdown of p23 expression in HeLa cells increases endogenous sAPPα secretion

**Figure 4:**
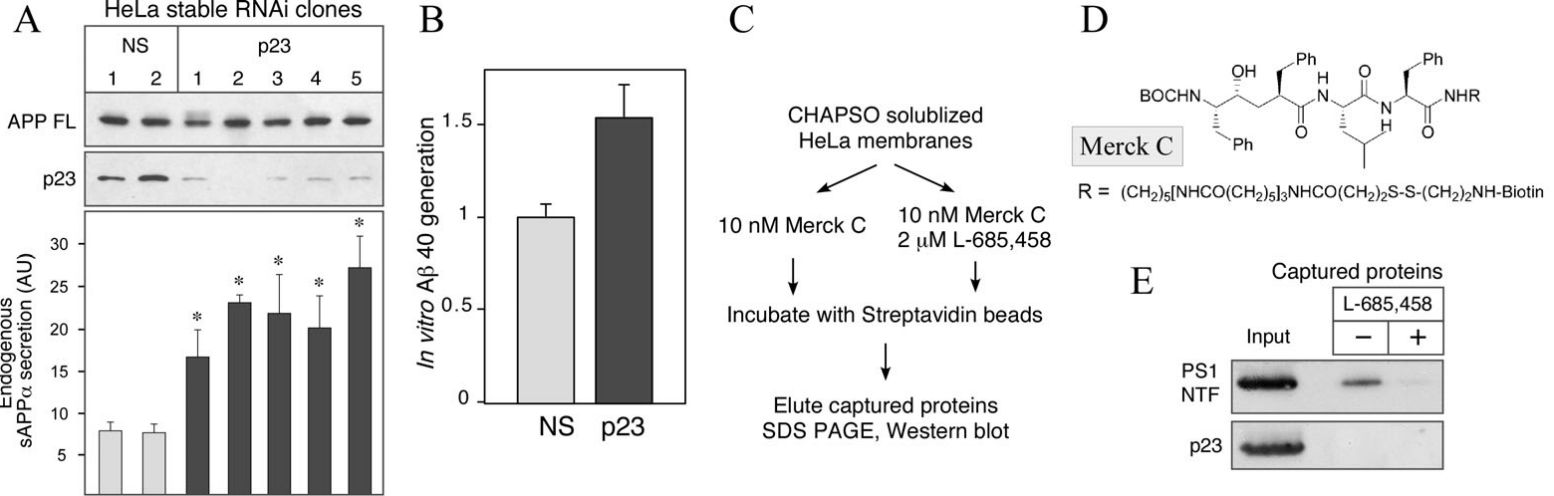
**p23 knockdown APP maturation and secretion in HeLa cells**. *A*, Lysates of HeLa cell lines stably transfected with p23 or control shRNA plasmids were probed with antibodies specific for APP and p23. Quantitative analysis of sAPPα in the media conditioned by each stable cell line was performed using ELISA. The data represent mean ± SEM of three sets of samples; *p *< 0.001. *B*, Membranes prepared from two control and four p23 knockdown HeLa cell lines were incubated *in vitro *with C100 FLAG substrate and Aβ_40 _generation was quantified by ELISA. *C*, The strategy for capturing active γ-secretase using the active-site γ-secretase inhibitor Merck C. *D*, The structure of Merck C. *E*, Solubilized HeLa S3 cell membranes were incubated with biotinylated γ-secretase inhibitor Merck C in the absence or presence of excess L-685,458. Active γ-secretase complexes were captured using streptavidin beads and analyzed by immunoblotting.

The data described above were obtained from the analysis of N2a or HeLa cells stably overexpressing FAD-linked mutant APPswe. It is known that secretase processing of wild type APP and APPswe are quite different with respect to the subcellular sites of proteolysis. For example, the presence of "Swedish" mutation renders APPswe extremely susceptible to cleavage by β-secretase in the biosynthetic organelles such as the Golgi and TGN [29]. Furthermore, because p24 family proteins have a well-established role in early secretory pathway trafficking of transport vesicles, p23 regulation of overexpressed APPswe could markedly differ relative to endogenous APP. To confirm increased secretion of sAPP from endogenous wild type APP, we generated HeLa cell lines stably transfected with non-specific control or p23 shRNA plasmids. This later RNAi approach was also effective in stably reducing expression of p23 in independent clones (Fig. 4A). By assaying conditioned media we found that each of the p23 knockdown clones displayed a significant increase in the secretion of endogenous sAPPα relative to clones transfected with the non-specific shRNA plasmid, while the steady-state levels of full-length APP (APP FL) remained unchanged by stable attenuation of p23 expression (Fig. 4A).

To address whether p23 knockdown influences cell-free generation of Aβ in HeLa cell membranes, we incubated C100FLAG substrate with membranes prepared from control and p23 knockdown clones using a well-established *in vitro *assay [30]. To demonstrate the specificity in these assays, one set of each sample was incubated in parallel with the γ-secretase inhibitor L-685,458 to inhibit Aβ production. By ELISA analysis, we found that membrane preparations from p23 knockdown clones generated about 50% more Aβ_40 _relative to membranes from control clones (Fig. 4B). We then asked whether p23 is incorporated into active γ-secretase complexes. To this end, we used Merck C, a specific biotinylated transition-state γ-secretase inhibitor that is capable of capturing active γ-secretase complexes from membrane preparations [31]. Interestingly, we found that the γ-secretase protein complexes captured from CHAPSO solubilized HeLa cell membranes contained readily detectable levels of PS1 NTF, but not p23 (Fig. 4E). As expected, we also detected PS1 CTF and nicastrin in γ-secretase complexes isolated using Merck C (data not shown). Moreover, addition of excess transition-state analog inhibitor L-685,458 prevented the capture, confirming selective isolation of active γ-secretase complexes by Merck C (Fig. 4E). These results indicate that p23 is not present at 1:1 stoichiometry with PS1 as a stable component of active γ-secretase complexes. Nevertheless, knockdown of p23 expression can influence *in vitro *Aβ production in a cell-free assay using an exogenous APP substrate and membrane preparations from HeLa (our results) or HEK293 cells [8], consistent with modulation of Aβ production by transient interaction of p23 with the mature γ-secretase complex.

### Role of p23 in PS1 FAD mutation-associated selective increase of Aβ_42 _secretion

**Figure 5:**
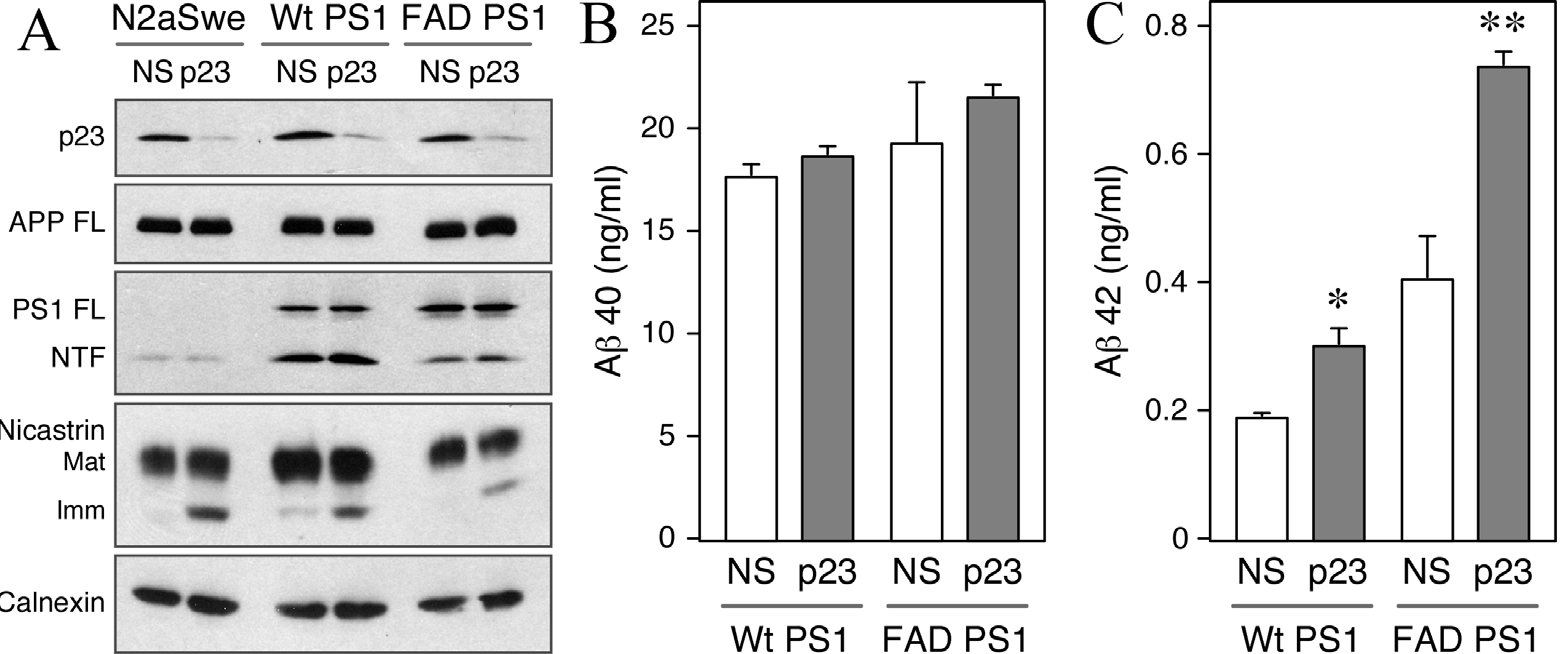
**Additive effects of p23 depletion and FAD-linked PS1 mutation on APP metabolism**. *A*, Lysates of N2a*Swe *cells stably expressing wt PS1 or C410Y mutant were probed with antibodies specific for p23, APP, γ-secretase components, and calnexin. *B *and *C*, Quantitative analysis of secreted Aβ_40 _and Aβ_42 _in the media conditioned by wt and C410Y cell lines was performed using two-site ELISAs. The data represent mean ± SEM of three transfections. * *p *< 0.05; ** *p *< 0.01.

FAD-linked mutations in PS1 and PS2 selectively elevate the levels of Aβ_42 _peptides by mechanism(s) that is still not clearly understood [32]. In contrast to FAD-linked mutations, knockdown of p23 expression affected multiple aspects of APP metabolism including biosynthetic stabilization of nascent APP and increased secretion of sAPP, Aβ_40 _and Aβ_42 _as described above. Therefore, we predicted that p23 modulation of Aβ_42 _production might be indirect, and distinct from the mechanism associated with selective FAD-linked PS1 mutant effect on Aβ_42 _generation. To test this idea, we performed p23 siRNA knockdown studies in N2a*Swe *derivatives co-expressing APPswe and either wt PS1 or a FAD-linked PS1 variant (C410Y) (Fig. 5A). Knockdown of p23 expression in cells overexpressing either wt or FAD-linked mutant PS1 had no effect on the levels of secreted Aβ_40 _peptides (Fig. 5B). The implication of this finding is not readily apparent, but might be the result of potential interaction between excess holoPS1 present in these cells. Nevertheless, wt PS1 cells transfected with p23 siRNA secreted significantly higher levels of Aβ_42 _relative to cells transfected with non-specific siRNA (0.19 ng/ml in NS siRNA cells *versus *0.30 ng/ml in p23 siRNA cells; *p *< 0.05) (Fig. 5C). As expected, the C410Y PS1 cells used in these experiments secrete 2-fold higher levels of Aβ_42 _relative to wt PS1 cells (0.19 ng/ml wt PS1 cells *versus *0.41 ng/ml C410Y cells; *p *< 0.05), but secrete comparable levels of Aβ_40 _(Fig. 5B and 5C). Moreover, cells expressing C410Y PS1 secreted even higher levels of Aβ_42 _following transfection with p23 siRNA (0.41 ng/ml in NS siRNA cells *versus *0.74 ng/ml in p23 siRNA cells; *p *< 0.001) (Fig. 5C). These data imply that p23 depletion has little influence on Aβ_40 _production in the presence of excess PS1 polypeptides. Furthermore, presence of FAD-linked PS1 mutation and p23 knockdown have additive effects on Aβ_42 _production, consistent with independent regulation of Aβ_42 _production by these two factors.

### Influence of p23 knockdown on the localization of APP and Mint3

**Figure 6:**
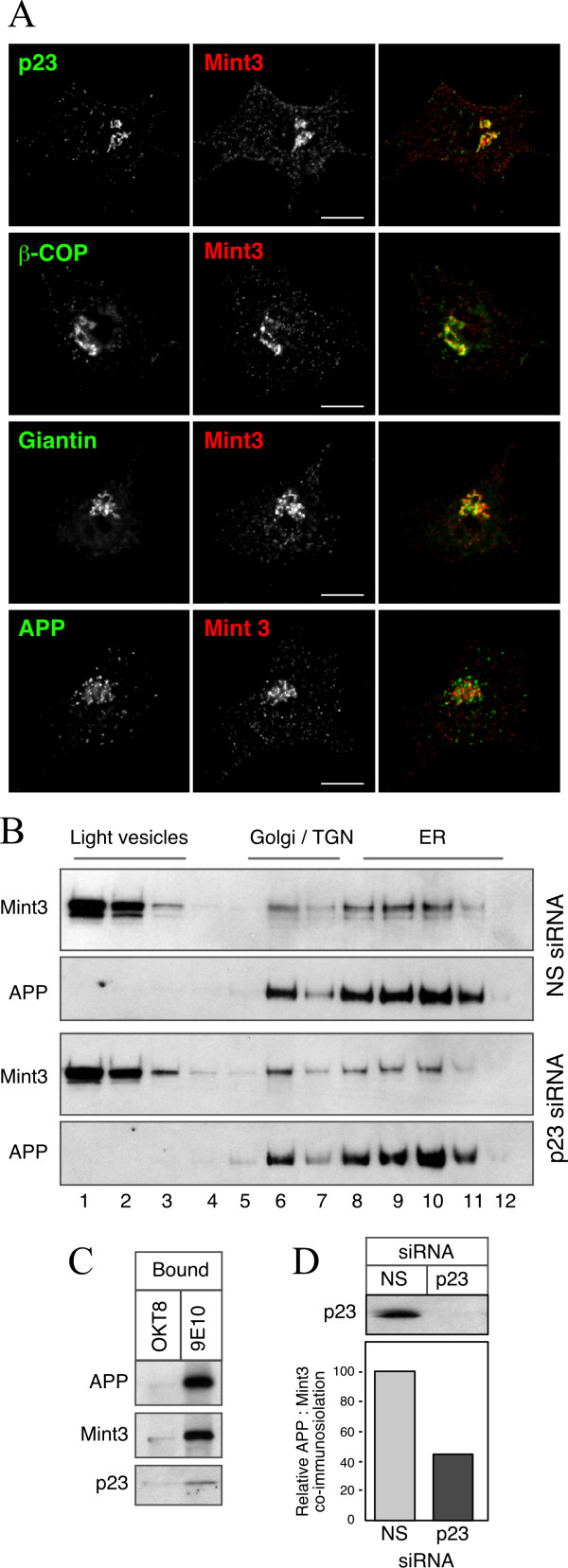
**p23 depletion affects the co-residence of APP and Mint3**. *A*, N2a cells transfected with control or p23 siRNA were analyzed by immunofluorescence staining with a mAb against APP adaptor Mint3 and polyclonal p23, β-COP, giantin, or APP antibodies. Bar represents 10 μm. *B*, siRNA transfected N2a*Swe *cells were lysed using a ball-bearing homogenizer and fractionated by velocity sedimentation. Equal volume aliquots of fractions were analyzed by immunoblotting using antibodies against Mint3 and APP. *C*, Aliquots of fractions 6–12 were pooled and incubated with magnetic beads coated with mAb OKT8 (negative control) or mAb 9E10 (recognizes C-term myc tag in APP). Bound membranes and vesicles were solubilized in Laemmli buffer and analyzed by SDS-PAGE and Western blotting. *D*, Immunoisolation was performed using N2a*Swe *cells transfected with control or p23 siRNA and relative signal intensity of co-immunoisolated Mint3 relative to APP was quantified and graphically represented.

How might p23 function affect sAPP secretion? p23 is an abundant protein in cis-Golgi and ERGIC membranes, and plays an important role in biosynthetic protein transport [22]. Despite earlier evidence that suggested a cargo receptor function for p24 family members [33], endogenous p23 does not readily co-immunoprecipitate with or cross-link to APP in N2a*Swe *cells, formally excluding the possibility that p23 modulates APP trafficking by direct protein interaction (data not shown). We then turned our attention to potential indirect mechanism(s) that might account for the observed redistribution of APP in cells transfected with p23 siRNA. Cytosolic adaptors such as members of Munc18-interacting protein (Mint) [also referred to as X11], Fe65, Dab, and JIP family of proteins regulate Aβ production by influencing APP trafficking and γ-secretase processing [34]. Mint3 (X11γ), the ubiquitously expressed Mint family member, is particularly interesting because it binds to ARF1, localizes to the Golgi, and can function as coat proteins to regulate APP trafficking [35]. Therefore, we asked whether p23 might influence APP trafficking by affecting Mint3 function. First, we visualized the distribution endogenous Mint3 and APP in N2a cells by immunofluorescence labeling. Consistent with previous descriptions, Mint3 and APP were concentrated in a juxtanuclear location, and showed considerable overlap with COPI coatomer subunit β-COP and the Golgi marker giantin (Fig. 6A). Furthermore, when N2a cells were disrupted without the use of detergents using a ball-bearing homogenizer and fractionated through a sucrose step gradient to separate organelles based on their density, we found co-fractionation of APP, Mint3 and p23 with membranes enriched in Golgi, TGN and ER makers (Fig. 6B). Note that Mint3 found in fractions 1–3 represents Mint3 associated with light-density vesicles, cytosolic Mint3, and Mint3 that had detached from Golgi membranes during cell lysis. The relative distribution of APP and Mint3 in sucrose density gradients was not markedly altered by p23 depletion. To obtain direct evidence for co-residence of APP, Mint3 and p23, we carried out antibody-mediated immunoisolation of APP containing vesicles and membrane organelles using magnetic beads coated with mAb 9E10 (which reacts with the C-terminal c-myc epitope tag of APPswe). Subsequent Western blot analysis revealed the presence of endogenous Mint3 and p23, supporting the notion that APP, Mint3 and p23 co-reside in early secretory compartments (Fig. 6C). Interestingly, p23 depletion caused marked diminution in the co-residence of Mint3 in membranes immunoisolated using magnetic beads coated with mAb 9E10 (Fig. 6D). These results show that p23 function likely influences co-residence of APP and its adaptor Mint3, providing a plausible mechanism by which p23 can affect APP trafficking and sAPP secretion independently of a direct APP-p23 interaction.

## Discussion

**Figure 7:**
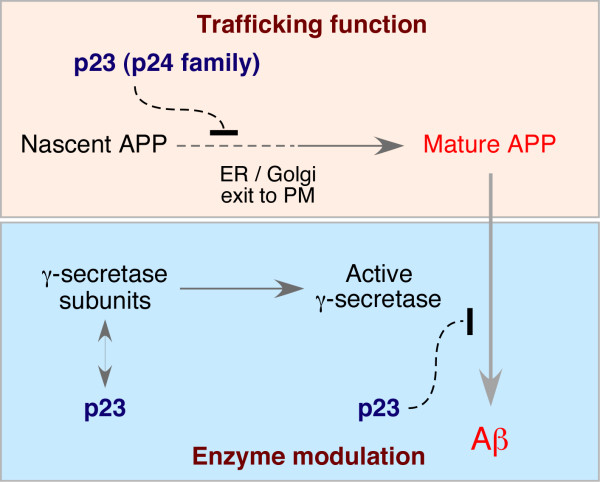
**Model depicting the dual role of p23 in APP trafficking and processing**. Our results suggest that p23 has dual roles in the negative regulation of APP metabolism. First, the function of p23/p24 family proteins in early secretory trafficking of proteins at the ER-Golgi boundary influences APP biogenesis in a manner that stabilizes nascent APP, as well as enhances APP maturation and steady-state accumulation at the cell surface. Second, p23 can modulate Aβ production by influencing the proteolysis of APP CTF by modulation of γ-secretase. Cells transfected with p23 siRNA secrete increased levels of Aβ. Moreover, absence of p23 expression also results in increased Aβ generated by proteolysis of recombinant APP CTF substrate in cell free assays, providing strong support for the notion that this latter function occurs independent of vesicular trafficking.

The main finding of our work is that p23/TMP21, and likely p24 family proteins, regulate APP biosynthesis and trafficking. siRNA knockdown of p23 expression enhances biosynthetic stability of nascent APP, and leads to enhanced maturation and cell surface accumulation of APP. Moreover, our study confirms that p23 can modulate Aβ production as recently reported [8], and documents that p23 modulation of Aβ_42 _production is independent of FAD mutant PS1-associated increase in γ-secretase cleavage of APP at the Aβ_42 _site. The efficiency of Aβ production is determined by multiple cellular events that control the trafficking and sequential proteolytic processing of APP. Despite remarkable progress on the characterization of APP secretases, the mechanisms that modulate cell surface transport of APP, especially during transit of nascent APP polypeptides through early secretory compartments, are not well understood. Because Aβ is mainly generated in late secretory and endocytic compartments [36], positive or negative control of biosynthetic APP transport in the ER and Golgi could significantly impact on the efficiency of amyloidogenic processing. Based on previous findings and our analysis of APP metabolism in p23-depleted cells reported here, we suggest that p23 has dual roles in the regulation of APP metabolism: modulation of γ-secretase processing of APP CTF [8]; and regulation of APP biogenesis and trafficking of APP holoprotein, which can further influence Aβ production (Fig. 7).

Members of the p24 family proteins cycle between cis-Golgi and the ERGIC [10]. Proposed functions of p24 proteins in ER/Golgi transport include integral receptors for COPI and COPII coatomer components, recruitment of ARF1 to Golgi membrane, Golgi organization, *de novo *formation of vesicular tubular clusters, generation of ER exit sites, and the formation of tubular transport intermediates [13,14,17,20,24,27,37]. Deletion of yeast p24 ortholog EMP24 impaired retention of Kar2p/BiP, indicative of defects in Golgi to ER retrieval [21]. Moreover, partial defects in Golgi maturation of Gas1p (a glycosyl-phosphatidylinositol anchored plasma membrane protein) and secretion of invertase (a soluble protein that is secreted into the periplasmic space) in yeast p24 deletion mutants led to the suggestion that p24 proteins regulate cargo entry into COPII vesicles [19,21]. Nevertheless, secretion of carboxypeptidase Y and pro α-factor were unaffected in yeast lacking all p24 proteins, arguing against an essential role in vesicular transport in the exocytic pathway [19]. Consistent with this idea, our [^35^S]met/cys labeling studies reveal only minor decrease in overall protein biogenesis or secretion in N2a cells upon p23 knockdown.

Based on p23 antibody-mediated inhibition of vesicular stomatitis virus G protein trafficking, it was proposed that p23 plays an obligatory role in forward biosynthetic transport of transmembrane proteins in mammalian cells [22]. In contrast, loss of SEL-9 (p24 ortholog) function in *C. elegans *increased cell surface trafficking of mutant but not wt GLP-1 (Notch homolog). Our data demonstrate enhanced APP maturation and cell surface accumulation in cells transfected with p23 siRNA. Moreover, we find an increase in the secretion of endogenous wt APP as well as stably overexpressed FAD-associated mutant APPswe following attenuation of p24 family protein expression. These findings are consistent with improved biosynthetic transport of APP to the cell surface in the absence of p23/p24 family function. In this regard, it is notable that PS1 deficiency also results in enhanced release of APP-containing vesicles from the ER and TGN, leading to increased maturation and cell surface accumulation of APP [39]. Considering that packaging of APP and PS1 into COPII vesicles during ER export is uncoupled [38], it is highly likely that p23 depletion affects APP FL trafficking and PS1-dependent γ-secretase activity *via *distinct mechanisms. In agreement, we find that expression of FAD-linked mutant PS1 and depletion of p24 proteins have additive effects on Aβ_42 _production. One plausible mechanistic explanation for our results is that p23 function restricts biosynthetic trafficking of APP; p23-dependent APP retention/retrieval mechanisms operating in the continuum of bidirectional transport between the ER and Golgi are compromised upon p23 knockdown, allowing enhanced transit of nascent APP from the ER/Golgi through the exocytic pathway. Thus, our study provides the important insight that p23 functions in both positive and negative regulation of transmembrane cargo transport in the early secretory pathway. Elucidating the precise function of p23 in controlling the fidelity of APP trafficking remains a challenge because overexpression of p23 leads to retention of exogenous and endogenous p24 proteins in the ER, causing experimental artifacts (data not shown) [10,24].

Several cytosolic adaptors with phosphotyrosine-binding domains, including Mint family proteins, Fe65 family members, and Dab1 bind to the APP cytoplasmic tail at or near the YENPTY motif, and regulate APP trafficking and processing [34]. Mint proteins can directly bind to ARFs in a GTP-dependent manner, and siRNA knockdown of Mint3 resulted in diffuse localization of APP throughout the cell and concomitant decrease in juxtanuclear Golgi concentration [35]. Thus it was postulated that Mints might serve as coat proteins in regulating vesicular trafficking of APP. We find that endogenous Mint3 localizes to the Golgi apparatus and shows colocalization with p23, β-COP and giantin. Depletion of p23 markedly reduced juxtanuclear concentration of Mint3 and membrane co-localization of APP and Mint3 evidenced by immunofluorescence and magnetic immunoisolation analyses (Fig. 6 and data not shown). Together, these results raise the intriguing possibility that Mint proteins may functionally link p23 to APP cargo and influence it's trafficking. Since Mints are recruited to the Golgi membrane by interaction with ARFs [35], and recruitment of ARF1 to the Golgi is mediated by p23 [17], it is reasonable to hypothesize that p23 influence on Mint3 localization may involve ARFs. This prediction awaits direct experimental confirmation. Molecular characterization of functional interactions between p23/ARF/Mint/APP is a focus of our future investigation. Finally, it is interesting to note that while the phosphotyrosine-binding domain of Mint proteins bind to APP cytosolic tail, the PDZ domains of Mint also bind to the C-terminus of PS1. Thus, functional interaction between p23/Mint/PS1 may be critically involved in the regulation of APP trafficking as well as γ-secretase processing.

## Methods

### Plasmids, oligonucleotides, and antibodies

Complementary oligonucleotides corresponding to three p23 target sequences separated by a 9-nucleotide non-complementary spacer (TTCAAGAGA) were synthesized and cloned into a modified pSUPER plasmid [40]. One of the three siRNAs directed against a conserved sequence 5'-ATACCTGACCAACTCGTGA (nt 416–434 of NM_006827) present in human and mouse *p23 *mRNA, was identified as the most efficient by Western blotting and used for subsequent experiments. Synthetic siRNA duplexes against the above sequence were purchased from Dharmacon. A non-specific control siRNA was designed against the sequence CTGCAGAGCTCGACCACTC [41]. The non-specific control shRNA plasmid was generated using oligonucleotides targeted to green fluorescence protein sequence.

Polyclonal p23 antisera were generated against a synthetic peptide ISFHLPVNSRKCLREEIHKDLLVTGA, corresponding to the N-terminal luminal residues 32–57 of mouse p23. Polyclonal Ab R8666 was raised against a synthetic peptide corresponding to the C-terminal 13 amino acids of APP and affinity purified. mAb B436 reacts with the amino-terminal region of Aβ and also recognizes sAPPα. mAb B113 and A387 were raised against Aβ sequences and are selective for Aβ_40 _and Aβ_42_, respectively. Antibodies against γ-secretase components [42], and p24 family proteins p24, p25, and tp24 have been described [12]. APP C-terminal Ab 369 was provided by Drs. Sam Gandy (Farber Institute for Neurosciences, Philadelphia) and Huaxi Xu (The Burnham Institute, La Jolla). Polyclonal GRASP55 antiserum was a gift of Vivek Malhotra (University of California, San Diego). The following antibodies were purchased from commercial sources: mAb P2-1 against APP N-terminus (Biosource), mAb 5228 (Chemicon), calnexin (Stressgen), β-COP (Affinity Bioreagents, Inc.), giantin (Covance), p115 and Mint3 (BD Biosciences), OKT8 and 9E10 (ATCC).

### Cell culture

Mouse N2a neuroblastoma cell lines expressing human APP695_SWE _and human PS1 have been previously described [29,43]. HeLa/APP_SWEDISH _cells were provided by Gang Yu (UT Southwestern, Dallas) [44]. HeLa cells were maintained in DMEM supplemented with 10% fetal bovine serum, penicillin/streptomycin, and 2 mM L-glutamine (Invitrogen). N2a cells were maintained in the medium above mixed 1:1 with OptiMEM (Invitrogen). For transient p23 knockdown, cells were transfected twice with 200 or 300 nM p23 siRNA in 60 mm dishes and experiments were performed 72 h later. HeLa cells were stably transfected with pSUPERp23neo and the efficacy of p23 depletion in individual clones was determined by Western blot analysis.

### Protein analyses

Total cell lysates for immunoblotting were prepared in IP buffer (50 mM Tris pH 7.4, 150 mM NaCl, 0.5% sodium dexoycholate, 0.5% NP40, 0.25% SDS, 5 mM EDTA, 0.25 mM PMSF, and supplemented with a protease inhibitor cocktail [Sigma]). Metabolic labeling using [^35^S]met/cys and APP immunoprecipitation were performed as described [29]. APP was immunoprecipitated from lysates and conditioned media using Ab 369 and mAb P2-1, respectively. Total protein synthesis and secretion were determined by using trichloroacetic acid precipitation combined with scintillation counting or by SDS/PAGE combined with phosphorimager detection. To measure cell surface APP, live cells were washed in cold HEPES buffer and blocked in HEPES buffer containing 0.1% BSA for 30 min at 4°C. Cells were then incubated at 10°C with mAb P2-1 for 90 min. After washing, cells were incubated for 3 h at 4°C with 1 μCi/ml ^125^I-conjuated anti-mouse secondary Ab (Amersham Biosciences). After extensive washes, cells were lysed in IP buffer and Ab binding was quantified by γ-counting. Non-specific binding was determined by omitting primary Ab, and specific binding was normalized to total protein concentration determined by BCA assay.

### Aβ and sAPPα measurements

Fresh medium was added to cells 24 h after transfection with siRNA and conditioned media were collected at 48 h. The levels of secreted Aβ_40_, Aβ_42_, and sAPPα were quantified using specific sandwich ELISAs. Briefly, 96-well white ELISA plates were coated with the appropriate capture mAb (B113 for Aβ_40_, A387 for Aβ_42_, and 5228 for sAPPα). Following sample incubation for all three ELISAs, plates were washed and Aβ_40_, Aβ_42 _or sAPPα were detected with alkaline phosphatase-conjugated mAb B436 and CSPD-Sapphire II Luminescence Substrate (Applied Biosystems). Each sample was assayed in duplicate using appropriate dilution of the conditioned media so that the relative luminescent units were in the linear range of the standards included on each plate. For Aβ quantifications synthetic Aβ_40 _and Aβ_42 _peptides were diluted in culture medium to generate standard curve. sAPPα sample relative luminescence unit values were compared to a standard curve prepared from affinity-purified sAPPα. Briefly, conditioned medium from cells expressing human wild-type APP751 was collected and passed over an affinity column linked with a mAb that recognized sAPPα but not sAPPβ. Fractions were eluted with low pH and neutralized. A pooled sample containing the sAPPα peak was quantified by a protein assay and utilized for the sAPPα standard curve.

### *In vitro *γ-secretase assays

Cell-free assays using C100FLAG substrate were performed essentially as described [30] using membranes prepared from non-specific control and p23 knockdown clones. Merck C, a highly specific biotinylated γ-secretase inhibitor [31], was used for affinity isolation of γ-secretase. The CHAPSO solubilized HeLa S3 membranes were incubated with 10 nM of Merck C for 2 h at 37°C in the absence or presence of 2 μM L-685,458. The samples were then incubated with streptavidin beads to capture inhibitor-bound γ-secretase complexes as previously described [31]. Bound proteins were released by incubating in SDS-sample buffer and analyzed by immunoblotting.

### Subcellular fractionation studies

Confluent cells from nine 60 mm dishes were homogenized using ball-bearing homogenizer with a 12 μm clearance and postnuclear supernatants were fractionated on sucrose density gradients essentially as described previously [42]. Twelve 1 ml fractions were collected from the top of the gradient using a fractionator and 60 μl of each fraction was analyzed by Western blotting. Immunoisolation of APP containing vesicles using magnetic beads coated with mAb 9E10 was performed essentially as described [42]. Antibody OKT8 was used as the negative control to establish the specificity of the immunoisolation procedure.

### Immunofluorescence microscopy

Cells cultured on poly-lysine coated coverslips were processed for immunofluorescence analysis as previously described [42]. Primary antibodies were diluted in PBS containing 3% BSA and 0.2% Tween-20 and added to fixed cells at room temperature for 2 h. Images were acquired as 200 nm *z*-stacks on a motorized Nikon TE2000 microscope with Cascade II:512 CCD camera (Photometrics, Tucson, AZ) using 100× 1.45 NA Plan-Apochromat oil objective. Images were deconvolved using Huygens software (Scientific Volume Imaging BV, The Netherlands) and processed using Metamorph software (Molecular Devices Corporation, Downingtown, PA).

## Abbreviations

Aβ, β-amyloid; APP, amyloid precursor protein; APP FL, full-length APP; ARF1, ADP ribosylation factor 1; COP, coat protein; CTF, C-terminal fragment; ER, endoplasmic reticulum; ERGIC, ER/Golgi intermediate compartment; FL, full-length; NTF, N-terminal fragment; PS, presenilin(s); shRNA, short hairpin RNA; TGN, trans-Golgi network.

## Authors' contributions

KSV, PG, JWB, and YC carried out the p23 knockdown and APP metabolism studies. KSV and HC carried out the Mint interaction experiments. KSV, PG and GT carried out the immunofluorescence staining experiments. LP and Y-ML performed the *in vitro *γ-secretase experiments. PDN and MZK performed the ELISA. KSV, PG, JWB, FTW, Y-ML, and MZK participated in data analysis and helped to draft the manuscript. GT conceived of the study, designed experiments, coordinated data analysis and prepared the manuscript. All authors read and approved the final manuscript.
